# Brain disease research based on functional magnetic resonance imaging data and machine learning: a review

**DOI:** 10.3389/fnins.2023.1227491

**Published:** 2023-08-17

**Authors:** Jing Teng, Chunlin Mi, Jian Shi, Na Li

**Affiliations:** ^1^School of Control and Computer Engineering, North China Electric Power University, Beijing, China; ^2^Department of Hematology and Critical Care Medicine, The Third Xiangya Hospital of Central South University, Changsha, China; ^3^Department of Radiology, The Third Xiangya Hospital of Central South University, Changsha, China

**Keywords:** brain diseases, functional magnetic resonance imaging, machine learning, diagnosis, feature selection

## Abstract

Brain diseases, including neurodegenerative diseases and neuropsychiatric diseases, have long plagued the lives of the affected populations and caused a huge burden on public health. Functional magnetic resonance imaging (fMRI) is an excellent neuroimaging technology for measuring brain activity, which provides new insight for clinicians to help diagnose brain diseases. In recent years, machine learning methods have displayed superior performance in diagnosing brain diseases compared to conventional methods, attracting great attention from researchers. This paper reviews the representative research of machine learning methods in brain disease diagnosis based on fMRI data in the recent three years, focusing on the most frequent four active brain disease studies, including Alzheimer's disease/mild cognitive impairment, autism spectrum disorders, schizophrenia, and Parkinson's disease. We summarize these 55 articles from multiple perspectives, including the effect of the size of subjects, extracted features, feature selection methods, classification models, validation methods, and corresponding accuracies. Finally, we analyze these articles and introduce future research directions to provide neuroimaging scientists and researchers in the interdisciplinary fields of computing and medicine with new ideas for AI-aided brain disease diagnosis.

## 1. Introduction

The brain is the most complicated and delicate biological organ in human cognition, which contains nearly 100 billion neurons with over 1,000 trillion synaptic connections between neurons (Koch and Laurent, 1999; Azevedo et al., 2009; Zhang, 2019). It processes various information humans obtain daily, regulates various bodily functions, and manages advanced activities such as emotion, movement, learning, and memory (Raji et al., 2009; Shoeibi et al., 2023). Due to the extremely fine biological structure of the brain, minor damage to its internal functions is highly likely to lead to diseases such as Alzheimer's disease (AD) (Tanveer et al., 2020), mild cognitive impairment (MCI) (Fathi et al., 2022), schizophrenia (SCZ) (Fathi et al., 2022), Parkinson's disease (PD) (Li and Li, 2022), autism spectrum disorders (ASD) (Moridian et al., 2022), and traumatic brain injury (Abdelrahman et al., 2022). Brain diseases, including neurodegenerative diseases and neuropsychiatric diseases, have long plagued the affected populations' lives and caused a huge burden on public health (Vardi and Merrick, 2008; Chen et al., 2022). However, there is currently no gold standard for diagnosing brain diseases worldwide due to the unclear pathogenesis of these diseases (de Lau and Breteler, 2006; Scheltens et al., 2016; Lord et al., 2018). Brain disease diagnosis mainly depends on the clinical symptom scores and doctors' experience in clinical practice, which is relatively subjective and inefficient, leading to some extent of misdiagnosis and omissions (Insel and Cuthbert, 2015; Wang et al., 2022a; Allely et al., 2023). How to accurately diagnose brain diseases has become a hot topic in many fields, such as psychology, medicine, and artificial intelligence.

Neuroimaging technologies, including functional magnetic resonance imaging (fMRI) (Logothetis, 2008), structural MRI (Wattjes, 2011), positron emission tomography (Nasrallah and Dubroff, 2013), diffusion tensor imaging (DTI) (Bihan et al., 2001), and electroencephalography (Mulkey et al., 2015), are important tools for studying and diagnosing brain diseases (Perrin et al., 2009; Abi-Dargham and Horga, 2016). Among many imaging modes, fMRI provides a new window for researchers to study dynamic brain activity observation and internal activation states (van den Heuvel and Pol, 2010). It detects the metabolic activity of the brain by measuring variations in blood flow and blood oxygen concentration, indirectly revealing distinctions in brain activity between patients and normal people, thereby providing new perspectives into the disease pathogenesis (Heeger and Ress, 2002; Slobounov et al., 2011; Birur et al., 2017). Researchers have proposed various imaging features based on fMRI in recent years to explore variations in brain function, such as the amplitude of low-frequency fluctuation (ALFF) (Zang et al., 2007), and regional homogeneity (ReHo) (Zang et al., 2004). These imaging metrics have different characteristics and advantages, and their emergence and development can help understand diseases' neurophysiological mechanisms from multiple perspectives.

**Figure 1:**
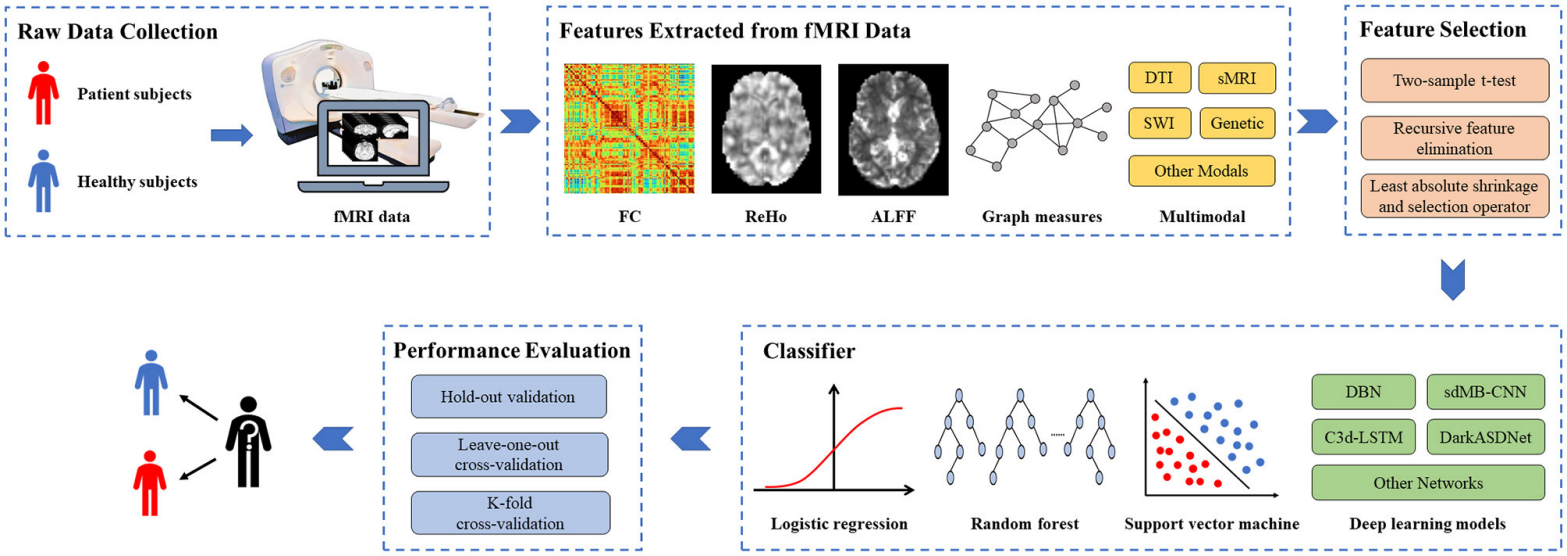
The general process for diagnosing brain diseases based on fMRI and machine learning. fMRI, functional magnetic resonance imaging; FC, functional connectivity; ReHo, Regional homogeneity; ALFF, Amplitude of low-frequency fluctuation; DTI, diffusion tensor imaging; sMRI, structural MRI; SWI, susceptibility-weighted imaging.

Researching AI-assisted diagnosis algorithms for brain diseases can alleviate the supply and demand contradiction between the limited number of doctors with professional diagnostic qualifications and the increasing number of disease patients, which could also improve the accuracy of diagnosis and treatment. Based on fMRI data, researchers study potential differences between patients and healthy subjects by machine learning methods, which have been extensively adopted in researching brain diseases such as AD, SCZ, MCI, and ASD. Although machine learning-based studies for brain disease classification surged recently, a comprehensive and detailed review of these studies is lacking. We searched for articles published in the recent three years, from January 1, 2020, to March 1, 2023, through the keywords “machine learning,” “fMRI,” “brain disease,” and “diagnosis” on the Web of Science. According to the title, abstract, and full-text content, 88 articles above the Journal Citation Reports Quartile 2 in the category were selected. According to the number of papers on brain diseases studied, the most frequent four active brain disease studies, including AD/MCI, ASD, PD, and SCZ, were selected, with a total of 55 papers. The general process for diagnosing brain diseases based on fMRI and machine learning is shown in Figure 1. We summarize the detailed information of these studies, such as data sources, dataset sizes, and extracted features in Table 1. Furthermore, the representative feature selection, machine learning, and evaluation methods are described in detail to help readers understand the benefits and usage of distinct methodologies. Finally, we summarize the current challenges and possible future research directions in this area, hoping to provide new perspectives for studying AI-aided diagnosis of brain diseases.

**Table 1:** Summary of research on the classification of brain diseases based on machine learning and functional magnetic resonance imaging data.

**References**	**Target**	**Subjects**	**Dataset**	**Modality**	**Extracted features**	**Atlas**	**Feature selection**	**Classification model**	**Validation**	**Accuracy**
Li et al. (2020a)	AD vs. MCI	116 AD, 99 MCI, 174 NC	ADNI	fMRI	4D fMRI data	N/A	N/A	C3d-LSTM	Hold-out	92.1
	MCI vs. NC									88.1
	AD vs. NC									97.4
	AD vs. NC vs. MCI									89.5
Zhang et al. (2020)	MCI vs. NC	82MCI, 93NC	ADNI	rs-fMRI	Neuropsychological assessments, network metrics	AAL	RF	RF	Nested CV	91.4
Kam et al. (2020)	eMCI vs. NC	49 eMCI, 48 NC	ADNI	rs-fMRI	Static BFNs, dynamic BFNs	N/A	N/A	sdMB-CNN	5-fold CV	76.1
Bi et al. (2020)	AD vs. NC	37 AD, 35NC	ADNI	rs-fMRI, SNP	ROI-gene pairs	AAL	CERF	SVM	Hold-out	86.2
Wang et al. (2020)	AD vs. NC	154 NC, 165 eMCI, 145 lMCI, 99 AD	ADNI	rs-fMRI	BOLD signals	AAL	N/A	STNet	5-fold CV	90.3
	lMCI vs. eMCI									79.4
	AD vs. MCI vs. NC									71.8
	AD vs. lMCI vs.									60.7
	eMCI vs. NC									
Li et al. (2020b)	MCI vs. NC	45 MCI, 46 NC	HCP,	rs-fMRI	FBN based NERTL	AAL	*t*-test	SVM	LOOCV	82.4
			NITRC							
Lei et al. (2020)	SMC vs. NC	38 lMCI, 44 eMCI, 44 SMC, 44 NC	ADNI	rs-fMRI	d-HON	AAL	Local weight clustering coefficient, LASSO	SVM	Nested CV	78.9
	eMCI vs. NC									80.3
	lMCI vs. NC									85.2
	lMCI vs. eMCI									78.8
	lMCI vs. SMC									84.3
	eMCI vs. SMC									80.2
Jin et al. (2020)	AD vs. MCI vs.	252 AD, 221 MCI,	INTERNAL	rs-fMRI	ReHo, FC,	Brainnetome	Meta analysis	SVM	LOSOCV	89
	NC	215 NC			AM, FC strength					
Xu et al. (2020)	MCI vs. NC	39 MCI, 60 NC	INTERNAL	rs-fMRI	FC, Graph measures	Power	*t*-test, gLASSO	MK-SVM	LOOCV	92.9
		27MCI, 23NC	ADNI							66.0
Liu et al. (2020b)	eMCI vs. NC	105 eMCI, 105 NC	ADNI	rs-fMRI, T1w MRI	GMV, SPL, non-imaging phenotypic measures	AAL	MTFS-gLASSO	GCN	5-fold CV	84.1
Liu et al. (2020a)	lMCI vs. NC	105 eMCI, 105 lMCI, 105 NC	ADNI	rs-fMRI, T1w MRI	GMV, CT, CC, SPL	AAL	MTFS-gLASSO-TTR	MK-SVM	Nested CV	88.5
	eMCI vs. NC									82.7
	lMCI vs. eMCI									79.6
Guo et al. (2020)	AD vs. NC	30 AD, 30 NC	ADNI	rs-fMRI	Graph measures	13 seed ROI	RF	RBK_SVM	10-fold CV	95.9
Guo and Zhang (2020)	MCI vs. NC	91 MCI, 79 NC	ADNI	rs-fMRI	FC	AAL	N/A	Autoencoder	10-fold CV	94.6 (Sens)
Bi et al. (2021a)	eMCI vs. NC	42 eMCI, 38 lMCI, 36 NC	ADNI	rs-fMRI	Graph measures	AAL	N/A	CEWSVME	Hold-out	83.5
	eMCI vs. lMCI									84.3
Lama and Kwon (2021)	MCI vs. NC	31 MCI, 31 NC, 31 AD	ADNI	rs-fMRI	Feature vector	AAL	LASSO	Linear SVM	10-fold CV	98.9
	AD vs. NC									90.6
	MCI vs. AD									97.8
Wang et al. (2021)	AD vs. NC	34 AD, 18 eMCI, 18 lMCI, 50 NC	ADNI	fMRI, sMRI	FC, gray matter images	AAL	PCANet, 3DShuffleNet	SVM	Hold-out	96.0
	AD vs. MCI									100.0
	MCI vs. NC									100.0
	lMCI vs. eMCI									100.0
Jia et al. (2021)	SMC vs. NC	34 AD, 26 SMC, 57 eMCI, 35 lMCI, 38 MCI, 50 NC	ADNI	fMRI	mALFF, mReHo	N/A	Improved 3DPCANet	SVM	N/A	91.3
	SMC vs. MCI									95.0
	SMC vs. AD									83.3
	eMCI vs. lMCI									85.2
	MCI vs. AD									86.4
	MCI vs. NC									88.9
	AD vs. NC									92.0
Bi et al. (2021b)	eMCI vs. NC	37 eMCI 36 NC	ADNI	rs-fMRI, genetic data	ROI-gene pairs	AAL	N/A	GERF	Hold-out	86.2
Zhang et al. (2021)	MCIc vs. MCInc	55MCInc, 30MCIc, 19AD	ADNI	rs-fMRI, sMRI	Cortical thickness features, structural brain network features, sub-frequency functional brain network features	Desikan -Killiany, AAL	RSFS	SVM	Nested CV	84.7
MCIc vs. AD	89.8
Gullett et al. (2021)	stable aMCI vs. declining aMCI	41 stable aMCI, 14 declining aMCI	INTERNAL	rs-fMRI, T1 sMRI	FC, T1 voxelwise intensity values	Yeo atlas	*t*-test	SVM	Nested CV	94.5
Bi et al. (2022)	eMCI vs. lMCI	37 eMCI, 26 lMCI	ADNI	rs-fMRI, genetic data	ROI-gene pairs	AAL	WERF	SVM	Hold-out	88.9
Zhou et al. (2022)	MCI vs. NC	42 MCI, 54 NC	INTERNAL	rs-fMRI, DTI	Significant HIP-related WM network	Seed based	RFE	SVM rbf	Hold-out	89.4
Wang B. et al. (2022)	AD vs. eMCI vs. lMCI vs. HC	30 AD, 34 lMCI, 53 eMCI, 43 HC	ADNI	rs-fMRI	Dynamic FC	HCP MMP	K-means	DBCP	Hold-out	86.0
Cai et al. (2022)	aMCI vs. HC	33 aMCI, 34 HC	ADNI	rs-fMRI	ReHo, ALFF, FC	AAL	MGS-WBC, VGBN-LM	SVM	LOOCV	94.0
Lu et al. (2022)	AD vs. NC	100 AD, 100 NC	ADNI	fMRI	FC	AAL	KFS-ELM	ELM	Hold-out	99.2
Zhang et al. (2022)	MCI vs. NC	66 MCI, 51 NC	ADNI	rs-fMRI	Multiatlas-based FCNs	AAL	RSGL	SVM	LOOCV	85.5
Ghafoori and Shalbaf (2022)	sMCI vs. pMCI	188 sMCI, 78 pMCI	ADNI	rs-fMRI	Functional images, clinical features	N/A	N/A	3D-CNN	5-fold CV	87.6
Khatri and Kwon (2022)											AD vs. NC	63 AD, 68 NC, 37 sMCI, 45 MCIc	ADNI	rs-fMRI, sMRI	Graph measures, Voxel Features, Hippocampus and Amygdala Volume	AAL	JMI	SVM	10-fold CV	95.9
	AD vs. MCI	92.5																	
	NC vs. MCI	90.4																	
	sMCI vs. MCIc	88.0																	
Alorf and Khan (2022)	CN vs. SMC	182 CN, 36 SMC, 213 eMCI, 145 lMCI, 14 MCI, 116 AD	ADNI	rs-fMRI	FC	AAL	N/A	SSAE network	10-fold CV	92.8
										86.8
	CN vs. eMCI									96.7
										87.8
	CN vs. MCI									90.9
										77.1
	CN vs. lMCI							BC-GCN	5-fold CV	94.9
										91.5
	CN vs. AD									97.0
										91.7
	CN vs. SMC vs. eMCI vs. MCI vs. lMCI vs. AD									94.2
										84.0
Mousa et al. (2022)	AD vs. NC	167 NC, 102 eMCI, 129 lMCI, 114 AD	ADNI	rs-fMRI	CorrTF matrix	AAL	*t*-test	SVM	10-fold CV	99.3
										AD vs. MCI	99.7
											NC vs. MCI	98.2
												eMCI vs. lMCI	100.0
													AD vs. NC vs. eMCI vs. lMCI	98.2
Lin et al. (2022)		AD vs. NC	99 AD, 145 lMCI, 165 eMCI, 154 NC	ADNI	rs-fMRI	dynamic FC	AAL	N/A	CRNN				5-fold CV	92.8
	eMCI vs. NC	84.5											
	AD vs. IMCI vs. eMCI vs. NC	61.7											
Ji and Yao (2021)	ASD vs. NC	527 ASD, 569 NC	ABIDE	rs-fMRI	FC	AAL	N/A	CNNGLasso	5-fold CV	68.3		
Liang et al. (2021)	ASD vs. NC	511 ASD, 561 NC	ABIDE	rs-fMRI	FC	CC200	Kendall rank correlation estimation	CNNPL	10-fold CV	76.0
Huang et al. (2021)	ASD vs. NC	505 ASD, 530 NC	ABIDE	rs-fMRI	FC	CC200	GBFS	DBN	10-fold CV	76.4
Subah et al. (2021)	ASD vs. NC	402 ASD, 464 NC	ABIDE	rs-fMRI	FC	BASC	N/A	DNN	5-fold CV	87.9
Haweel et al. (2021)	ASD vs. NC	50 ASD, 50 NC	NDAR	Task-fMRI	BOLD signals	HO	K-means, DWT	2D-CNN	10-fold CV	80.0
Ahammed et al. (2021)	ASD vs. NC	79 ASD, 105 NC	ABIDE	fMRI	2D images	N/A	N/A	DarkASDNet	N/A	94.7
Cao et al. (2022)	ASD vs. NC	539 ASD, 573 NC	ABIDE	rs-fMRI	Dynamic graphs	CC200	N/A	GSA-LSTM	10-fold CV	68.4
Hu et al. (2022)	ASD vs. NC	13 ASD, 21 NC	ABIDE	rs-fMRI	FC	AAL	N/A	FCG-MTGS-TSK	10-fold CV	71.4
		18 ASD, 16 NC								59.1
		15 ASD, 20 NC								75.2
Wang et al. (2022b)	ASD vs. NC	479 ASD, 478 NC	ABIDE	rs-fMRI	FC	AAL	N/A	Random SVM cluster	Hold-out	88.1
Zhao et al. (2022)	ASD vs. NC	48 ASD, 50 NC	ABIDE	rs-fMRI	FC	Dosenbach atlas	Boruta	SVM	LOOCV	92.9
Wang N. et al. (2022)	ASD vs. NC	280 ASD, 329 NC	ABIDE	rs-fMRI	FC	BASC	SIMLR, Nested SVD	SVM	LOOCV	68.4
Liang et al. (2022)	ASD vs. NC	480 ASD, 509 NC	ABIDE	rs-fMRI	FC	CC200	N/A	MSA-DNN	10-fold CV	70.5
ElNakieb et al. (2023)	ASD vs. NC	408 ASD, 476 NC	ABIDE	rs-fMRI	dynamic FC	AAL	F-Score, RFECV	linear SVM	5-fold CV	98.8
Cao et al. (2020)	PD vs. NC	68 PD, 48 NC	INTERNAL	rs-fMRI, sMRI	mALFF, mReHo, FC, VMHC, GM, clinical information	HO	Mann Whitney U test, removing features with high correlation, LASSO	SVM	10-fold CV	84.8
Pang et al. (2021)	TD vs. PIGD	57 TD, 39 PIGD	INTERNAL	rs-fMRI	mReHo, mALFF, DC, VMHC, FC	AAL	*t*-test, LASSO, Spearman's rank correlation	SVM	Hold-out	87.5
Shi et al. (2021b)	PD vs. NC	48 PD, 32 NC	Public database	rs-fMRI	Intensity-based histogram features of the ALFF	AAL	*t*-test, LASSO	Rag-score	10-fold CV	93.8
		11 PD, 9 NC								90.0
Shi et al. (2022)	PD vs. NC	59 PD, 41 NC	Public database	rs-fMRI	ALFF-based Radiomic features	Brainnetome	*t*-test, LASSO	SVM	Nested CV	81.5
		27 PD, 16 NC	FCP/INDI							67.4
Pang et al. (2022)	MSA-P vs. IPD	77 IPD, 75 MSA-P	INTERNAL	rs-fMRI, DTI, T1 images, SWI	Iron-radiomic features, function, diffusion, volumetric measures, clinical variables	Brainnetome	mRMR, LASSO	SVM	Hold-out	93.4
Chen et al. (2023)	MSA-P vs. IPD	76 MSA-P, 53 IPD	INTERNAL	rs-fMRI	Graph measures, FC	AAL	*t*-test, RF, removing features with high autocorrelation	LR	Hold-out	92.3
Yang et al. (2020)	SCZ vs. NC	236 SCZ, 150 NC	INTERNAL	Task-fMRI	Graph measures	Power	N/A				SVM	Hold-out	71.6
Ghosal et al. (2021)	SCZ vs. NC	43 SCZ, 54 NC	INTERNAL	Task-fMRI, SNP	Imaging, Genetics	Brainnetome	N/A	Generative- discriminative framework	10-fold CV	73.0
Shi et al. (2021a)	SCZ vs. NC	45 SCZ, 64 NC	COBRE	rs-fMRI, sMRI	ALFF, ReHo, DC, VMHC, GMD	Brainnetome	*t*-test	MLDA	LOOCV	83.5
				Zang et al. (2021)	SCZ vs. NC						61 FESCZ, 79 CSCZ, 205 NC	INTERNAL	rs-fMRI, sMRI	GMV, ReHo, ALFF, DC	GWB	PCA	LR	LOOCV	88.0
FESCZ vs. CSCZ	RFE	86.0																
Wang T. et al. (2022)	SCZ vs. NC	60 SCZ, 71 NC	COBRE	rs-fMRI	FC	AAL	N/A	MKCapsnet	10-fold CV	82.4

N/A indicates information was not available or could not be found.

INTERNAL denotes subjects were recruited from institutional and/or public through media channel.

AD, Alzheimer's disease; MCI, mild cognitive impairment; NC, normal control; ADNI, Alzheimer's disease Neuroimaging Initiative; fMRI, functional magnetic resonance imaging; LSTM, long short-term memory; rs-fMRI, resting state fMRI; AAL, Automated Anatomical Labeling; RF, random forest; CV, cross-validation; eMCI, early MCI; BFNs, brain functional networks; CNN, convolutional neural network; sdMB-CNN, multiple-static and dynamic BFNs-Based CNN; SNP, single nucleotide polymorphism; ROI, region of interest; CERF, cluster evolutionary RF; SVM, support vector machine; lMCI, late MCI; BOLD, blood-oxygen-level-dependent; STNet, Spatial-Temporal convolutional-recurrent neural Network; HCP, human connectome project; NITRC, Neuroimaging Informatics Tools and Resources Clearinghouse; FBN, functional brain network; NERTL, Network Estimation via Regularized Transfer Learning; LOOCV, leave-one-out CV; SMC, significant memory concern; d-HON, dynamic high-order functional network; LASSO, least absolute shrinkage and selector operator; ReHo, regional homogeneity; FC, functional connectivity; AM, the amplitude of local brain activity; LOSOCV, leave-one-site-out CV; gLASSO, group LASSO; MK-SVM, Multi-kernel SVM; GM, gray matter; GMV, GM volume; SPL, shortest path length; MTFS-gLASSO, multi-task feature selection-gLASSO; GCN, graph convolutional network; CT, cortical thickness; CC, clustering coefficient; MTFS-gLASSO-TTR, MTFS-gLASSO-task-task relationship; RBK_SVM, SVM with radial basis kernel function; CEWSVME, clustering-evolutionary weighted SVM ensemble; sMRI, structural MRI; PCANet, Principal component analysis network; mALFF, mean ALFF; mReHo, mean ReHo; GERF, genetic evolutionary RF; MCInc, MCI non-converters; MCIc, MCI converters; RSFS, random subset feature selection algorithm; aMCI, amnestic MCI; WERF, weighted evolutionary RF; DTI, diffusion tensor imaging; HIP, hippocampus; WM, white matter; RFE, recursive feature elimination; rbf, radial basis function; HC, healthy control; HCP MMP, human connectome project multimodal parcellation; DBCP, dynamic brain connectivity processing method; MGS-WBC, the mask generation strategy based on within-class and between-class criterion; VGBN-LM, vector generation for brain networks based on Laplacian matrix; ELM, Extreme Learning Machine; KFS-ELM, Key Features Screening Method based on ELM; RSGL, relationship-guided sparse group learning; sMCI, stable MCI; pMCI, progressive MCI; JMI, Joint Mutual Information; CN, cognitively normal; SSAE, Stacked sparse auto-encoders; BC-GCN, Brain Connectivity GCN; CorrTF, correlated transfer function; CRNN, convolutional recurrent neural network; ASD, autism spectrum disorder; ABIDE, Autism Brain Imaging Data Exchange; CNNGLasso, CNN-based model with graphical LASSO; CC200, Craddock 200; CNNPL, CNN With Prototype Learning; GBFS, the graph-based feature-selection; DBN, deep belief network; BASC, Bootstrap Analysis of Stable Clusters; DNN, deep neural network; NDAR, National Database for Autism Research; HO, Harvard Oxford; DWT, discrete wavelet transform; GSA-LSTM, graph structure-aware LSTM; FCG-MTGS-TSK, Feature-Correlation-Guided Multitask Group Sparse Takagi “Sugeuo” Kang Fuzzy Systems; SIMLR, similarity-driven multiview linear reconstruction; SVD, singular value decomposition; MSA-DNN, multi-scale attention-based DNN; PD, Parkinson's disease; VMHC, voxel-mirrored homotopic connectivity; PIGD, postural instability and gait difficulty; TD, tremor-dominant; DC, degree of centrality; Rag-score, radiomic signature score; FCP/INDI, Functional Connectomes Project/International Neuroimaging Data-Sharing Initiative; IPD, idiopathic PD; MSA-P, parkinsonian variant of multiple system atrophy; SWI, susceptibility-weighted imaging; mRMR, minimal redundancy maximal relevance; LR, logistic regression; SCZ, schizophrenia; COBRE, The Center for Biomedical Research Excellence; GMD, GM density; MLDA, maximum uncertainty linear discriminate analysis; FESCZ, first-episode SCZ; CSCZ, chronic SCZ; GWB, the groupwise whole-brain; MKCapsnet, multi-kernel capsule network.

## 2. Features extracted from fMRI data

As can be seen from Figure 1, extracting features from fMRI data is a crucial step for diagnosing brain diseases based on machine learning. The extracted features include functional connectivity (FC), ReHo, ALFF, and graph measures. Besides, multimodal features, such as iron-radiomic features from susceptibility-weighted imaging (SWI) and diffusion from DTI, are also adopted for brain disease diagnosis. These features are important for improving model performance and exploring the biological mechanisms of brain function.

### 2.1. Functional connectivity

FC describes the degree of correlation between blood-oxygen-level-dependent (BOLD) time series in two different brain regions, which has been demonstrated to be a vital tool for revealing various disease mechanisms (Price et al., 2014). The brain atlas divides the entire brain into multiple regions of interest (ROI) based on functional or structural definitions. The average BOLD time series for all voxels within the ROI coordinate ranges represent the BOLD time series for each ROI. FC is mainly obtained by computing the Pearson correlation coefficient of the BOLD time series between paired ROIs. The calculation formula is as follows:


(1) $${\text{FC}}_{i,j}=\frac{cov(X_{i},X_{j})}{S_{X_{i}}S_{X_{j}}},1\leq i,j\leq M,$$

where X_*i*_ represents *i*th ROI time series, *S* is the standard deviation, *cov* is the covariance function, and *M* denotes the number of ROIs in the atlas. Based on the calculated FC value, a symmetric functional connectivity network is established, represented by FCN = [FC_1, 1_, FC_1, 2_, ⋯ , FC${}_{M,M}]\in {\mathbb{R}}^{M\times M}$. The widely utilized atlas is the automatic anatomical labeling (AAL) atlas (Tzourio-Mazoyer et al., 2002; Guo and Zhang, 2020; Ji and Yao, 2021; Pang et al., 2021; Wang et al., 2021, 2022b; Alorf and Khan, 2022; Cai et al., 2022; Hu et al., 2022; Lu et al., 2022; Wang T. et al., 2022; Chen et al., 2023). Besides, FC can be constructed by other atlases, such as the Power atlas (Power et al., 2011; Xu et al., 2020), Craddock 200 atlas (Craddock et al., 2011; Huang et al., 2021; Liang et al., 2021, 2022), Bootstrap Analysis of Stable Clusters (Bellec et al., 2010; Subah et al., 2021; Wang N. et al., 2022), Brainnetome atlas (Fan et al., 2016; Jin et al., 2020), Yeo atlas (Yeo et al., 2011; Gullett et al., 2021), Harvard-Oxford atlas (Desikan et al., 2006; Cao et al., 2020), and Dosenbach atlas (Dosenbach et al., 2010; Zhao et al., 2022). In particular, Zhang et al. (2022) constructed multiple FCNs based on the selected set of the atlas from generated multiple personalized atlases from the AAL atlas to improve the diagnosis effect of MCI.

The above FC refers to the traditional static FC, which reveals the inherent similarity between a pair of ROIs or particular networks (Liu et al., 2021). Dynamic FC is mainly extracted from fMRI data based on the sliding window strategy, which is more helpful than static FC in understanding the basic dynamic characteristics of the human brain (Hutchison et al., 2013). For each subject with M ROIs, an appropriate size and a sliding step of the time window are set, then the time window is slid according to the step over the entire time series. Statistical correlation coefficients such as Pearson are used to calculate the FCN for each window. Continuous sliding and calculation can obtain a set of T FCNs. Dynamic FCN can be denoted as dFCN = [FCN^1^, FCN^2^,⋯ , FCN^*T*^] ∈ ℝ^*T*×*M*×*M*^, where *T* is the number of overlapping windows. Lin et al. (2022) constructed dFCNs based on the sliding window strategy from resting state fMRI (rs-fMRI) data and extracted advanced features of dFCNs to classify brain disease by the proposed convolutional recurrent neural network. Wang B. et al. (2022) constructed dFCNs based on human connectivity project multimodal partitioning. Different from static FCN, dFCN can reveal more useful information for distinguishing between patients with brain diseases and healthy subjects. ElNakieb et al. (2023) proposed an enhanced version of dFCN, where correlation calculations consider temporal dynamics. The accuracy of the presented dFCN achieved 5% higher than traditional FCN. Kam et al. (2020) extracted static brain functional networks (BFNs) from rs-fMRI data by the Group Information Guided Independent Component Analysis method and generated dynamic BFNs based on seed association and sliding window strategy. The experimental results indicated that the simultaneous use of static BFNs and dynamic BFNs prioritizes using static BFNs alone, as dynamic BFNs provide additional time information for BFNs. Li et al. (2020a) obtained better functional brain network estimates based on regulated transfer learning to identify MCI patients from normal controls.

### 2.2. Regional homogeneity

ReHo is a major tool for detecting local neural synchronization (Xing et al., 2021). It reflects the consistency of regional brain activity by computing Kendall's coefficient of concordance between a specific voxel and its neighboring voxels (Zang et al., 2004; Pang et al., 2021). The calculation formula is defined as follows:


(2) $$\begin{array}{r} ReHo=\frac{12\sum_{i=1}^{t}{\left(R_{i}-\bar{R}\right)}^{2}}{k^{2}(t^{3}-t)},\\ R_{i}=\sum_{j=1}^{k}r_{i,j},\\ \bar{R}=\frac{1}{t}\sum_{i=1}^{t}R_{i}, \end{array}$$

where *t* is the length of the BOLD time series, *k* is the number of local voxels studied, and r_*i, j*_ represents the ranking level of a specific voxel *j* in the entire time series under the *i*th moment. R_*i*_ denotes the rank sum of *k* voxels at the *i*th moment. $\bar{R}$ is the average value at all times. Mean ReHo (mReHo) is obtained by dividing the average ReHo value of the entire brain (Cao et al., 2020; Jia et al., 2021; Pang et al., 2021).

### 2.3. Amplitude of low-frequency fluctuation

ALFF is an effective fMRI data analysis tool widely used in various diseases to assess resting state activity in brain regions (Zhang et al., 2015; Pang et al., 2021; Zang et al., 2021; Cai et al., 2022). Firstly, the Fourier transform is performed on the time series of each voxel to obtain the frequency domain power spectrum. ALFF is obtained by calculating the square root of the power spectrum and taking the average value over a predefined frequency range (Zang et al., 2007). Mean ALFF (mALFF) is calculated by dividing the mean ALFF value of the entire brain (Cao et al., 2020; Jia et al., 2021; Pang et al., 2021). In addition, several researchers have broadened the range of brain disease classification characteristics from different perspectives. For example, Shi et al. (2021b) extracted intensity-based ALFF histogram features in 2021 to effectively discover regions of the brain with aberrant activity in PD patients. In 2022, Shi et al. (2022) extracted radiological features based on ALFF for the classification study of PD, providing further support for interpreting the PD's pathological mechanism.

### 2.4. Graph measures

As a branch of discrete mathematics, graph theory is frequently employed in network analysis of diverse brain diseases (Farahani et al., 2019; Hallquist and Hillary, 2019; Guo et al., 2020; Yang et al., 2020). The vertices *V* and edges *E* are the two most important concepts in constructing the graph *G*(*V, E*). Based on graph theory, the ROIs are regarded as vertices *V* and the connections between ROIs as edges *E*, thereby constructing a brain network graph for complex brain network analysis.

The metrics in complex brain network analysis mainly include nodal and global measures. Nodal measures characterize the nodal behavior of a network, such as the nodal shortest path length *L*(*i*), betweenness centrality *BC*(*i*), nodal local efficiency *E*_*loc*_(*i*), nodal efficiency *E*_*nodal*_(*i*), and nodal clustering coefficient *C*(*i*) (Freeman, 1977; Watts and Strogatz, 1998; Latora and Marchiori, 2001; Xu et al., 2020). Global measures indicators reflect the overall network organization, such as clustering coefficient ***C***_***P***_, characteristic path length ***L***_***P***_, small-worldness σ, local efficiency ***E***_***loc***_, and global efficiency ***E***_***glob***_ (Watts and Strogatz, 1998; Latora and Marchiori, 2001; Rubinov and Sporns, 2010). Table 2 summarizes the definitions of these nodal and global measures.

**Table 2:** The definitions of common nodal and global measures.

	**Measures**	**Definitions**
Nodal Measures of node *i*,
	denoted by *n*_*i*_
	(Freeman, 1977; Watts and Strogatz, 1998; Latora and Marchiori, 2001; Xu et al., 2020)	Nodal shortest path length *L*(*i*)	$L(i)=\frac{1}{N-1}\sum_{i\neq j\in V}d_{i,j}$, where *d*_*i, j*_ = *L*_*min*_(*n*_*i*_, *n*_*j*_), *N* is the number of nodes in graph *G*
	Nodal clustering coefficient *C*(*i*)	$C\left(i\right)=\frac{e_{i}}{D_{i}\left(D_{i}-1\right)/2}$, where *e*_*i*_ is the number of edges directly connected to *n*_*i*_, and *D*_*i*_ is the degree of *n*_*i*_
	Betweenness centrality *BC*(*i*)	$C(i)=\sum_{j\neq i\neq k\in V}\frac{\delta_{j,k}(i)}{\delta_{j,k}}$, where δ_*j, k*_ is the number of shortest paths from *n*_*j*_ to *n*_*k*_, and δ_*j, k*_(*i*) is the number of those shortest paths that pass through *n*_*i*_
Nodal local efficiency *E*_*loc*_(*i*)	$E_{loc}(i)=\frac{1}{N_{G_{i}}(N_{G_{i}}-1)}\sum_{j\neq k\in V_{G_{i}}}\frac{1}{d_{j,k}}$, where *G*_*i*_ is the subgraph composed of neighbors of *n*_*i*_
Nodal efficiency *E*_*nodal*_(*i*)	$E_{nodal}(i)=\frac{1}{N-1}\sum_{i\neq j\in V}\frac{1}{d_{i,j}}$
Global Measures (Watts and Strogatz, 1998; Latora and Marchiori, 2001; Rubinov and Sporns, 2010)	Clustering coefficient ***C***_***P***_	$C_{P}=\frac{1}{N}\sum_{i\in V}C(i)$
	Characteristic path length ***L***_***P***_	$L_{P}=\frac{1}{N}\sum_{i\in V}L(i)$
	Small-worldness **σ**	$\sigma =\frac{\gamma }{\lambda }$, where $\gamma =\frac{C_{P}}{C_{r}}$, $\lambda =\frac{L_{P}}{L_{r}}$, *C*_*r*_ denotes the random clustering coefficient, and *L*_*r*_ denotes the random characteristic path length
	Local efficiency ***E***_***loc***_	$E_{loc}=\frac{1}{N}\sum_{i\in V}E_{loc}(i)$
	Global efficiency ***E***_***glob***_	$E_{gloc}=\frac{1}{N}\sum_{i\in V}E_{nodal}(i)$

### 2.5. Multimodal

Some researchers diagnose disease by comprehensive classification features, integrating more information from various features. For example, Pang et al. (2021) extracted indicators such as ReHo, ALFF, and FC as classification features to provide more comprehensive information on brain function change. Chen et al. (2023) constructed graph measures and FC from rs-fMRI data based on the AAL atlas to distinguish the Idiopathic PD (IPD) and the parkinsonian variant of multiple system atrophy (MSA-P). Jia et al. (2021) extracted mReHo and mALFF from the Brainnetome atlas to distinguish MCI and AD, achieving an accuracy of 86.4%. Ghafoori and Shalbaf (2022) utilized clinical features and functional images extracted from rs-fMRI data to distinguish stable MCI and progressive MCI, with an accuracy improvement of 1.9% compared to using only functional images.

In recent years, multimodal analysis has also been widely adopted in the research of brain diseases by fusing the features of imaging data such as fMRI, structural MRI (sMRI), and DTI to identify more consistent biomarkers and explore the relationship between different modes (Liu et al., 2020b; Zhang et al., 2021; Khatri and Kwon, 2022). Gullett et al. (2021) achieved 94.5% accuracy in classifying stable amnestic MCI (aMCI) and declining aMCI by combining rs-fMRI and T1 sMRI modalities, which is better than only one modality. Shi et al. distinguished SCZ and healthy control (HC) by integrating multimodal information, including fMRI and sMRI. The results revealed that the most discriminate regions between SCZs and HCs mainly locate in the 15 regions, such as the left superior parietal lobule (Shi et al., 2021a). Pang et al. (2022) distinguished IPD and MSA-P by extracting iron-radiomic features, function, diffusion, and volumetric measures from SWI, rs-fMRI, DTI, and T1-weighted imaging, which achieves an accuracy of 91.1%. The classification accuracy was improved to 93.4% by combing clinical variables into the multimodal model. Cao et al. (2020) established a PD diagnosis framework by incorporating relevant clinical features, whole-brain functional connectivity and activity, and gray matter structure, which achieved 84.8% accuracy. The results found that unique whole-brain functional activity and connectivity in PD are mainly located in five networks, such as the affective network. These studies indicated the potential of multimodal data fusion in the auxiliary diagnosis of PD patients.

The fusion of fMRI and genetic data is another research method for multimodal analysis. Using imaging and genetic data to identify biomarkers and classify diseases can help reveal the pathogenesis of diseases, which is of great significance for the development of computational medicine (Bi et al., 2020, 2022; Ghosal et al., 2021). Ghosal et al. (2021) extracted Imaging and Genetics features from task-fMRI and single nucleotide polymorphism to identify SCZ patients from 97 subjects. Bi et al. (2020) constructed ROI-gene pairs based on rs-fMRI and genes to classify early MCI (eMCI) and late MCI (lMCI) patients. The discovery of the most discriminating ROIs and risk genes can provide essential bases for pathological research in the development of MCI.

In addition to the commonly used classification features mentioned above, rs-fMRI time series, 4D fMRI data, and 2D images loaded from fMRI data can be directly used as classification features (Li et al., 2020a; Wang et al., 2020; Ahammed et al., 2021). In particular, Lama and Kwon (2021) adopted the Node2vec graph embedding method to convert brain networks into feature vectors as input to the model. Mousa et al. (2022) calculated the intensity time series of each region and extracted the correlation transfer function (CorrTF) matrix by the CorrTF. It is a promising biomarker for early AD recognition, helping clinicians discover and investigate the affected brain regions and their potential associations during AD development.

## 3. Feature selection

The dimension of the above-extracted features usually exceeds the total number of samples, leading to the dimensionality curse (Liu et al., 2021; Khatri and Kwon, 2022). In addition, processing numerous features may resulting model overfitting. The feature selection step aims to preserve the features with strong correlation, improve model performance and minimize the computing cost. It plays a crucial role in correlation analysis, which could explain the affection of brain regions on classification performance. The following briefly describes the three most frequently used feature selection algorithms in Table 1.

### 3.1. Two-sample *t*-test

The two-sample *t*-test is a filtered feature selection method to compare whether each feature mean of two independent sample sets has significant differences (Liu et al., 2018). Two-sample *t*-test requires two groups of samples to be independent of each other and subject to normal distribution. The calculation formula that satisfies homoscedasticity is as follows:


(3) $$\begin{array}{l} t=\frac{\bar{X_{1}}-\bar{X_{2}}}{\sqrt{\frac{\left(n_{1}-1\right)S_{1}^{2}+\left(n_{2}-1\right)S_{2}^{2}}{n_{1}+n_{2}-2}\left(\frac{1}{n_{1}}+\frac{1}{n_{2}}\right)}}, \end{array}$$

where $\bar{X_{1}}$ and *S*_1_ represent the mean and standard deviation of each feature of the sample set, and *n*_1_ is the capacity of the sample set. The smaller the *t* value, the higher the significance level of the feature. The features are classified according to the calculated *t* value, and the features with weak discrimination ability are removed to improve the classifier's performance. Mousa et al. applied a *t*-test to select discriminative features between normal subjects and AD patients. Then they trained a support vector machine (SVM) with these features, achieving an accuracy of 99.3% (Mousa et al., 2022). Shi et al. (2021a) proposed a model obtaining an accuracy of 83.5%, which determined the different characteristics between the SCZ and health subjects by two-sample *t*-test and used maximum uncertainty linear discriminate analysis-based classifier.

### 3.2. Recursive feature elimination

Recursive feature elimination (RFE) is a wrapper feature selection approach that recursively reduces the size of the original feature set through multiple training rounds on a base model. SVM-RFE calculates the features' contribution to the model's classification performance by training SVM on the original feature set. Then, remove the features with small contributions and repeat the training process until the remaining features match the required number. Zhou et al. (2022) used the RFE to select and rank the features and revealed the pathological mechanism of MCI through the feature contribution ranking. Zang et al. (2021) reduced the dimensionality of features by RFE before the classification.

### 3.3. Least absolute shrinkage and selection operator

The least absolute shrinkage and selection operator (LASSO) is an embedded feature selection method, which is a least square method with an *L*_1_ penalty item (Hsu et al., 2008; Pang et al., 2021; Khatri and Kwon, 2022). LASSO calculates model coefficients β by minimizing the following functions:


(4) $$\begin{array}{l} \beta =\arg_{\beta }\min\left\{\overset{n}{\underset{i=1}{\sum }}(y_{i}-\overset{m}{\underset{j=1}{\sum }}\beta_{j}x_{ij}-\beta_{0}{)}^{2}+\alpha \overset{m}{\underset{j=1}{\sum }}|\beta_{j}|\right\}, \end{array}$$

where *n* is the total number of samples, *y*_*i*_ is the category label of the *i*th subject, *x*_*ij*_ is the *j*th characteristic of the *i*th subject, β_*j*_ represents the regression coefficient of the *j*th feature, and *m* is the feature dimension. α is a non-negative regularization parameter that aims to limit the sparsity of the model. When the bigger α is, the more β_*j*_ is set to zero to achieve the purpose of feature selection to reduce unimportant features. Shi et al. (2021b) applied a *t*-test (*P* < 0.05) and LASSO to determine the characteristics that significantly differed between PD patients and healthy subjects. Cao et al. selected features using the Mann–Whitney *U*-test, pre-determined thresholds to screen for highly correlated variables, and LASSO. These selected features were then used to distinguish PD from healthy subjects (Cao et al., 2020).

## 4. Classifier

Based on the selected features, the classifier distinguishes patients and healthy subjects. The classification accuracies are greatly impacted by the classifiers, summarized in Table 1.

### 4.1. Logistic regression

Logistic regression (LR) is a common method to solve the binary classification problem, which judges the category of samples by comparing the relationship between decision boundaries and samples. Its main idea is to assume that the data obey a certain distribution and make parameter estimation according to the maximum likelihood estimation. Chen et al. (2023) combined a *t*-test with random forest (RF) to screen out the discriminative features and then distinguish the IPD and MSA-P subjects by LR, obtaining 92.31% accuracy.

### 4.2. Random forest

RF is an ensemble classification model based on the decision tree, which can process high-dimensional data without feature selection. It randomly deletes some features in decision tree training, then selects features with better classification ability through integrated learning to improve the overall performance. In addition, Bi et al. (2020, 2021b, 2022) optimized the traditional random forest and successively proposed the cluster evolutionary RF, genetic-evolutionary RF, and weighted evolutionary RF algorithms based on genetic data and rs-fMRI data. Improved models can dynamically delete irrelevant or redundant sample features to discover the disease-related risk genes and brain regions.

### 4.3. Support vector machine

SVM is a powerful supervised machine learning approach aiming to find a decision boundary in the high-dimensional space that can maximize the interval between two categories. SVM has good generalization and robustness, which has advantages in solving small data sets and over-fitting problems in learning. Yang et al. (2020) diagnosed 236 SCZ patients from 386 subjects by SVM, achieving an accuracy of 71.6%. Gullett et al. (2021) applied SVM to separate stable participants from those with the greatest marginal decline at follow-up with an accuracy of 94.5%. Shi et al. (2022) built SVM to distinguish PD patients from normal subjects, obtaining 81.5% accuracy on the initial training set containing 100 subjects. Furthermore, Bi et al. (2021a) proposed the evolutionary weighted SVM ensemble method to investigate the changes from normal subjects to eMCI to lMCI.

The selection of kernel function and kernel parameter is crucial to SVM's performance. Unlike traditional SVM, multiple kernel SVM (MK-SVM) trains SVM by the kernel that fuses multiple kernel functions. It can effectively learn features of multi-mode data and is more flexible than single-kernel SVM. For example, Liu et al. (2020b) combine structural and functional features by MK-SVM to complete the MCI classification task. Xu et al. (2020) adapted MK-SVM to combine multimodal information to effectively distinguish MCI and healthy subjects, and accuracy was achieved at 92.9%.

### 4.4. Deep learning models

Besides the classifiers mentioned above, deep learning models have also been extensively adopted in brain disease diagnosis. With powerful learning and analysis capabilities, deep learning models can automatically find the representation with strong recognition and directly learn the optimal strategy from the original data using hierarchical structures of varying complexity. For instance, Kam et al. (2020) presented a new convolutional neural network (CNN) framework sdMB-CNN, which extracts deep embedded characteristics from static and dynamic functional connectivity networks through 3D-CNN. Compared with traditional methods such as SVM, it significantly improves the diagnosis performance by nearly 10%. Wang et al. presented the multi-kernel capsule network considering the brain's anatomical structure. Set the kernel to match the brain's anatomical structure partition size to capture the connection between regions on different scales (Wang T. et al., 2022). Research shows that the capsule network is viable and prospective in diagnosing SCZ. To directly process 4D fMRI data, Li et al. (2020a) designed a C3d-LSTM for AD recognition, which integrated 3D-CNNs and a long short-term memory network to capture the spatial and time-varying information in the data. Huang et al. (2021) designed a graph-based classification model based on the deep belief network, which is more accurate and efficient than other advanced methods. Ahammed et al. (2021) proposed the DarkASDNet model, predicting the binary classification between ASD patients and normal subjects by 3D fMRI data, with a classification accuracy of 94.7%.

## 5. Performance evaluation

As shown in Figure 1, the performance evaluation is the final step in brain disease diagnosis. Larson (1931) proposed in the 1930s that training and evaluating the performance of models on the same data would yield overly optimistic results. Accordingly, cross-validation (CV) is proposed to avoid overfitting by testing the model on new data sets, which can extract as much effective information as possible from limited data.

### 5.1. Hold-out validation

Hold-out validation randomly divides the original data set into independent training and test set (Arlot and Celisse, 2010). This method is simple to operate and avoids the overfitting problems caused by traditional training and testing based on the same data. However, different partitioning methods lead to different results, requiring multiple grouping to ensure the stability and persuasiveness of the model's result. Zhou et al. (2022) evaluated the model's classification performance by the hold-out method. They divided 80% of the dataset into the training set and the rest into the testing set. The dataset was randomly divided by the same proportion for evaluation 100 times. The model acquired an average classification accuracy of 89.4%. Wang et al. (2022b) evaluated the model performance by dividing the dataset into a training set, a validation set, and a testing set at the percentage of 40%, 10%, and 50%, respectively. The hold-out validation was repeated 50 times in their study.

### 5.2. Leave-one-out cross-validation

Leave-one-out CV (LOOCV) is the most classical exhaustive CV method, which selects one sample from the original data set as the test data, and the remaining are training data (Arlot and Celisse, 2010; Xu et al., 2020; Cai et al., 2022; Zhao et al., 2022). The experimental data are not affected by random factors because almost all samples in each round are applied to train the model. To avoid the problem of overfitting, Cai et al. (2022) adopted the LOOCV method to estimate the model's classification performance on a dataset containing 67 subjects to overcome the problem of limited data. The obtained results of LOOCV are stable and reliable, ensuring the reproducibility of the experimental process. However, it is more excessive computational overhead than other CV methods, suitable for limited sample sets.

### 5.3. K-fold cross-validation

K-fold CV was presented by Geisser (1975) as an alternative to the computationally expensive LOOCV, which divides the dataset into *k* subsets of almost equal size. Each subset of data is tested once, and the rest *k*-1 subsets are used as training sets to obtain k models. Combine and analyze the classification accuracy of *k* models to obtain more stable and accurate model performance. The constant *k* is variable. When *k* is the total number of samples in the dataset, k-fold CV is equivalent to LOOCV. Under limited samples, k-fold CV is the simplest and most extensively adopted method for evaluating model performance (Kam et al., 2020; Ghosal et al., 2021; Khatri and Kwon, 2022; Lin et al., 2022).

## 6. Research summary

### 6.1. Functional magnetic resonance imaging data sources

The articles listed in Table 1 mainly come from two fMRI data sources: internal datasets collected by self or cooperative agencies and public data sets. The former typically come from the same location and have the same collection parameters. The latter often comes from data from different locations, inevitably bringing heterogeneity to the dataset regarding functional magnetic resonance scanners, data collection protocols, and participant population differences, making disease research and analysis more challenging (Ghiassian et al., 2016). However, public datasets typically contain more available data than internal datasets, which can accommodate the demands of more academics for large-scale database analysis. Table 3 summarizes the status of papers and related datasets for the top four brain diseases in the past three years, with 81.8% of the articles using public data. Of these, 90.3% of AD/MCI studies and 100% of ABIDE were conducted on public datasets, demonstrating that well-maintained databases can advance research technology.

**Table 3:** Public dataset information and number of disease studies using public datasets.

**Disease**	**Number of studies**	**Studies based on public datasets**	**Public datasets**
MCI/AD	31	28 (90.3%)	Alzheimer's disease Neuroimaging Initiative^*a*^
			Human Connectome Project^*b*^
			Neuroimaging Informatics Tools and Resources Clearinghouse^*c*^
			ASD	13	13 (100%)	Autism Brain Imaging Data Exchange^*d*^
National Database for Autism Research^*e*^					
PD	6	2 (33.3%)	Functional Connectomes Project/International Neuroimaging Data-Sharing Initiative^*f*^
			Nanjing Brain Hospital public database^*g*^
			SCZ	5	2 (40%)	The Center for Biomedical Research Excellence^*h*^

^*a*^http://adni.loni.usc.edu/.

^*b*^https://www.humanconnectome.org/study/hcp-young-adult.

^*c*^http://www.nitrc.org/projects/modularbrain/.

^*d*^http://fcon_1000.projects.nitrc.org/indi/abide/.

^*e*^http://ndar.nih.gov.

^*f*^http://fcon_1000.projects.nitrc.org/indi/retro/parkinsons.html.

^*g*^http://dx.doi.org/10.6084/m9.figshare.1433996.

^*h*^http://fcon_1000.projects.nitrc.org/indi/retro/cobre.html.

### 6.2. Relationship between accuracy and sample size

**Figure 2:**
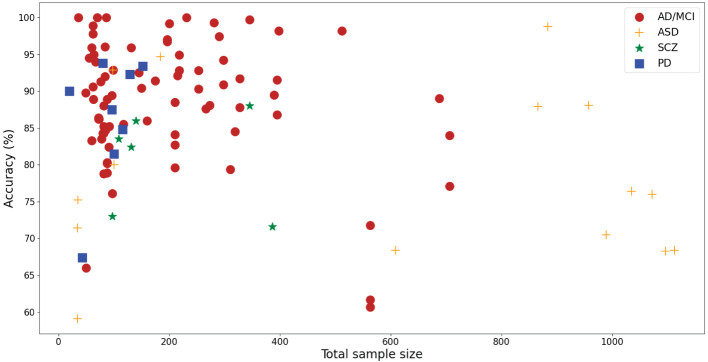
Scatter plot of the total sample size and reported accuracy.

Figure 2 shows the relationship between sample sizes and corresponding experimental accuracy. Among them, experiments with high accuracy (79.2% of experiments have an accuracy higher than 80%) are concentrated in the case of sample sizes less than 200 (60% of studies). It can be observed from Figure 2 that the overall trend of AD/MCI and SCZ is that accuracy declines with increasing sample size, which raises concerns about the generalization problem of small sample size research.

### 6.3. Distribution of the total sample size

**Figure 3:**
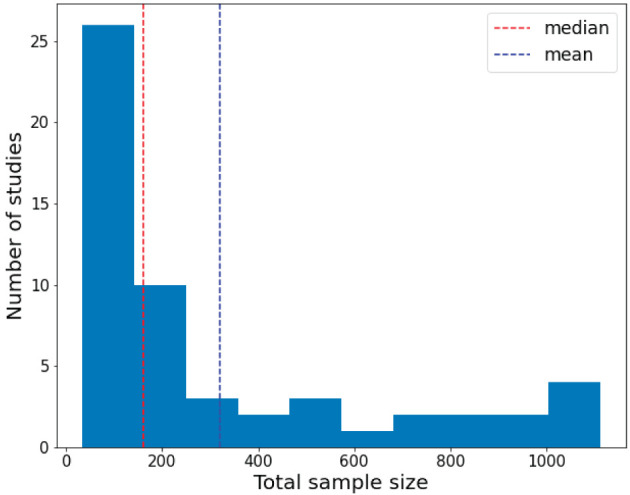
Distribution of the total sample size of the investigated papers.

Figure 3 displays the histogram of the sample sizes of investigated papers, which clearly illustrates the relationship between the overall sample size and the number of studies. The red dashed line denotes the average, while the blue dashed line represents the median. As depicted in Figure 3, the conducted research in the survey mainly focuses on small sample datasets due to the difficulty in sample collection in clinical practice.

### 6.4. Accuracy of each disease

**Figure 4:**
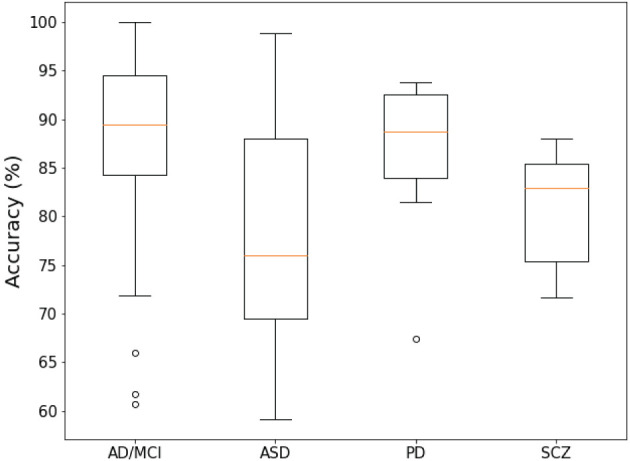
Box-plot of the overall accuracy of the report of the investigated papers.

Figure 4 shows a summary of statistical information on the overall accuracy of each disease report. The median accuracies of AD/MCI and PD studies exceed 85%. In contrast, the median accuracy of ASD is less than 80%. The median accuracy of SCZ is between 80 and 85.

### 6.5. Validation methods

**Figure 5:**
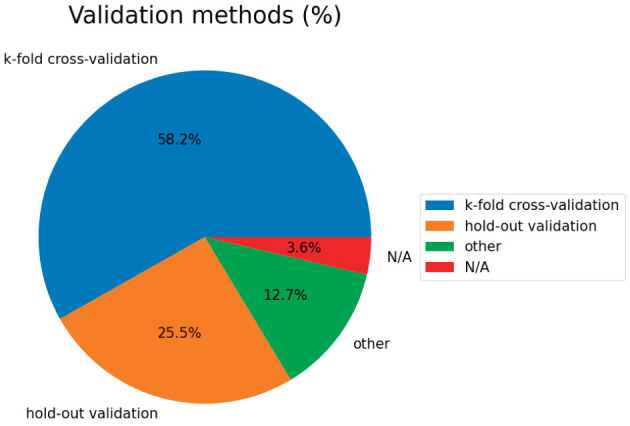
Validation methods of the surveyed studies.

Figure 5 shows the validation methods used in the papers. Regarding classifier verification methods, k-fold CV is the most commonly used method (accounting for 58.2%, 32/55 articles). Among them, the commonly used methods are LOOCV and 10-fold CV. Due to a lack of subjects, the hold-out scheme ranked second.

## 7. Summary and future research directions

This article reviews 55 brain disease classification studies based on fMRI data and machine learning in the recent three years. We summarize in detail these studies' dataset information, extracted features from fMRI, feature selection approaches, classification methods, and overall accuracy. In addition, different feature selection strategies and representative machine learning models are introduced, which can help researchers choose appropriate methods for practical classification problems. A new advancement in the classification of brain diseases based on fMRI data has been realized thanks to the quick development of machine learning technologies. Here, we propose five directions that researchers need to focus on, which are expected to promote the development of the AI-aided diagnosis of brain diseases.

1) Explore specific machine-learning methods for fMRI

Generating new machine learning technologies for the specific characteristics of fMRI by integrating the advantages of distinct methods to improve existing methods. We attempt to design specific machine learning methods to gather more beneficial information, thereby better identifying reliable biomarkers and assisting clinicians in diagnosis.

2) The application of fuzzy technology

The uncertainty of fMRI data, introduced by the instability of data collection equipment, the complexity of brain disease pathophysiology, and the fuzziness in determining medical causes, raises a challenge for brain disease diagnosis. Fuzzy technology, which can deal with the uncertainty of knowledge and expression, has been extensively adopted in the medical field (Parveen and Singh, 2015; Baskar et al., 2018; Chowdhary et al., 2020; Hu et al., 2022). Parveen and Singh (2015) segmented MRI images by fuzzy c-means clustering (FCM) to detect suspicious regions. Baskar et al. (2018) identified NC, MCI, and AD by combining kernel FCM and backpropagation artificial neural networks, which can improve classification performance by removing suspicious training samples. A new classification model based on Takagi-Sugeno-Kang fuzzy inference systems for ASD is proposed by Hu et al. (2022), which has been proven advantageous in interpretability and accuracy. Diagnosing brain diseases based on fuzzy technology could be a promising direction.

3) Multiple-feature fusion

Many studies have shown that various indicators may be complementary in revealing changes in brain function and can provide more beneficial information. For example, Pang et al. (2021) extracted multiple indicators based on fMRI data, including FC, mReHo, mALFF, degree centrality, and voxel-mirrored homotopic connectivity to classify PD motion subtypes by SVM. The results show that the multi-layer index combination of rs-fMRI can further promote performance. Kam et al. (2020) extracted deep embedding characteristics from static and dynamic BFNs through a designed 3D-CNN framework. This extension method considers the subtle and complex (spatiotemporal) changes of each BFN in eMCI and can use deep embedding features well. The mutual complementation of static and dynamic BFNs information further improves the diagnostic accuracy of eMCI. As a result, multiple-feature fusion is a valuable direction for future research, which could enhance existing research results.

4) Multimodal data fusion

Fusing multimodal data has shown notable prospects in analyzing changes in brain function at the individual level. For example, Gullett et al. (2021) combined T1w MRI and rs-fMRI to predict the overall model accuracy of diagnostic changes in aMCI reached 94.5%, significantly better than the method using only single mode diagnosis. In order to explore the potential of sMRI and rs-fMRI in AD recognition, Khatri and Kwon (2022) utilized their combined features in their research to achieve maximum classification accuracy relative to a single feature. In addition, Bi et al. (2020) fused fMRI and gene features to investigate the association between brain regions and genes, offering novel insights for the early detection and clinicopathological analysis of eMCI. Deep learning has potential advantages in processing complex data. Effectively combining multimodal data with deep learning models to improve diagnostic accuracy is a direction worth exploring in the future.

5) Establish an interpretable network

Deep learning models can be more accurate for classifying and identifying diseases than machine learning. However, the lack of interpretability of deep learning models has restricted their application in the clinical field. Improving deep learning models to establish interpretable networks are more conducive to understanding the brain distinctions between patients and healthy subjects, thereby benefiting neuroscientists studying brain diseases in the future.

## Author contributions

JT: supervision, conceptualization, and manuscript draft. CM: manuscript draft, conceptualization, and data analysis. JS: investigation. NL: investigation, data analysis, and manuscript draft. All authors listed have contributed substantially and directly to the article and approved it for publication.
